# Response of Red Sea phytoplankton biomass to marine heatwaves and cold-spells

**DOI:** 10.1038/s41598-025-88727-5

**Published:** 2025-02-11

**Authors:** Iason Theodorou, George Krokos, John A. Gittings, Sofia Darmaraki, Ibrahim Hoteit, Dionysios E. Raitsos

**Affiliations:** 1 Department of Biology, National and Kapodistrian University of Athens, 15772 Athens, Greece; 2 Institute of Oceanography, Hellenic Centre for Marine Research, 19013 Anavyssos, Greece; 3 Physical Science and Engineering Division, King Abdullah University of Science and Technology, Thuwal, 23955 Saudi Arabia

**Keywords:** Climate-change impacts, Ocean sciences, Climate-change ecology

## Abstract

In tropical oceans, phytoplankton experience significant alterations during marine heatwaves (MHWs), yet the consequences of reduced or absent marine cold-spells (MCSs) on these microscopic algae are currently overlooked. Synergistically combining *in situ* measurements, Argo-float data, remotely-sensed observations, and hydrodynamic model outputs, we explore such relationships in the Red Sea. Results show a long-term (1982 to 2018) gradual increase in MHW days (5–20 days/decade) and a clear decrease in MCS days (10–30 days/decade). Compound extreme temperature and chlorophyll-a events (Chl-a – an index of phytoplankton biomass) exhibit consistently lower Chl-a concentrations during MHWs and higher ones during MCSs, particularly in the northern and southern Red Sea. In these regions, during the main phytoplankton-growth period, the presence of MHWs/MCSs leads to respective Chl-a anomalies in 94% of the cases. Yet, phytoplankton responses in the central Red Sea are more complex, most likely linked to the region’s highly dynamic circulation (e.g., mesoscale anti-cyclonic eddies), and multiple nutrient sources. In the naturally warm and stratified ecosystem of the Red Sea, where deeper mixed layers enhance the transfer of nutrient-rich waters to the lit zone, the substantial reduction of MCSs could be more impactful for phytoplankton than the gradual rise of MHWs.

**Supplementary Information:**

The online version contains supplementary material available at 10.1038/s41598-025-88727-5.

## Introduction

Oceanic warming has been reported worldwide, over the past decades^1,2^, with severe implications for marine ecosystems^3^. In addition to this ongoing trend, ocean temperature extremes, such as marine heatwaves (MHWs) and marine cold-spells (MCSs), have recently been shown to impact marine ecosystems and the communities that rely on them^4,5^. MHWs are characterized by prolonged periods of abnormally warm water^6^, while MCSs manifest as discrete and prolonged episodes of unusually cold water^5^. Over the course of the 21st century, it is anticipated that MHWs will increase in frequency and intensity^7^, while the occurrence of MCSs is expected to decrease^5^. Despite the numerous studies examining the trends and characteristics of MHWs^7–9^ and MCSs^5,10–12^ at a global scale, few studies have investigated temperature extremes in marginal seas, such as the Mediterranean Sea^13–18^ or the Red Sea^19,20^. Thus, there is a crucial need for further investigation into the evolution and impacts of these extremes in marginal seas, which can serve as proxies for understanding the future of our oceans under different warming scenarios^21–23^.

The Red Sea is a rapidly warming Large Marine Ecosystem^24^ that has been subject to rising temperatures surpassing global warming rates^25–27^. The Red Sea’s ecological and economic significance is rooted in its rich marine biodiversity and extensive coral reef ecosystems^28–30^. Observations have revealed a significant increase in Sea Surface Temperature (SST) since 1994, attributed to both global warming^27^ and the positive phase of the Atlantic Multidecadal Oscillation (AMO) during recent decades^26^. Although a future negative phase of the AMO may temporarily mask or slow down the fast rate of increasing SST^26^, the long-term impacts of climate change are already evident in Red Sea coral reef ecosystems^31–34^. For instance, a direct link between coral bleaching events and MHWs in the Red Sea was revealed, highlighting the impact of these extremes on marine life at local scales^19^. Furthermore, the consequences of rising SST are also apparent in the open waters of the Red Sea, where changes in phytoplankton phenology and biomass have been reported^35,36^.

Phytoplankton are the cornerstone of primary production in the marine environment^37^. These microscopic algae play a crucial role in absorbing atmospheric CO_2_ and facilitating its transport to deeper waters^38–40^. The main limiting factors of phytoplankton growth are light and nutrient availability^41,42^. Particularly in temperate and tropical seas, nutrient availability is the main factor limiting primary production^43^. Generally, in such environments, vertical mixing during winter redistributes deeper, nutrient-enriched colder waters to the euphotic zone, increasing phytoplankton biomass^44–46^. In contrast, tropical oceans are often highly stratified and characterized by lower phytoplankton biomass, especially during summer when surface waters are considerably warmer^43,47^. Consequently, in such marine ecosystems, MHWs that may extend thermal stratification during the winter, inhibiting nutrient supply from deeper waters, have been linked to a decrease in phytoplankton biomass^48–51^, and/or changes in phytoplankton community structure^52^. Conversely, MCSs may drive more intense vertical mixing, leading to increased primary production^53,54^. The response of phytoplankton to MHWs has received increased attention in recent studies^9,49,51,55–61^, as opposed to research on the response to MCSs^5,53,62^. Indeed, studies that encompass both marine cold-spells and heatwaves, as well as their respective impacts on phytoplankton, are still scarce^54,63,64^.

The accessibility of open data from diverse *in situ* oceanographic platforms, modeled datasets, and remotely-sensed observations facilitates such studies. In particular, satellite-derived observations offer a cost-effective option to study large-scale alterations in oceanic surface temperatures, together with variations in chlorophyll-a concentration (hereafter Chl-a, a proxy for phytoplankton biomass)^65–68^. While satellite datasets provide a high sampling frequency and extensive temporal coverage of surface variables, *in situ* data and model outputs may provide insights into the dynamics of the entire water column. In fact, *in situ* data offer higher quality but limited spatiotemporal coverage, in comparison to model outputs that have adequate resolution, but lower accuracy. Given the significant influence of Mixed Layer Depth (MLD) variations and thermal stratification on nutrient availability^43–45,47^, it is important to integrate surface and subsurface ocean data to better comprehend extreme warming effects on phytoplankton biomass, especially in relatively unexplored tropical ecosystems, like the Red Sea^69^.

Combining satellite-derived observations, model outputs, research-cruise *in situ* data, and available Argo-floats, our study investigates the response of phytoplankton biomass to MHWs and MCSs across the entire Red Sea. We report the temporal trends of MHWs and MCSs (between 1982 and 2018) and the anomalous responses of phytoplankton biomass to temperature extremes (between 1998 and 2018). Finally, we provide an in-depth analysis of representative MHW or MCS cases and their corresponding influence on Chl-a concentrations.

## Results

The Red Sea was divided into four regions: the Northern Red Sea (NRS), North Central Red Sea (NCRS), South Central Red Sea (SCRS), and Southern Red Sea (SRS). This categorization is aligned with previous work on the spatiotemporal distribution of surface Chl-a in the basin^70^. Our analysis was centered around winter, which generally constitutes the main growth period of phytoplankton in the Red Sea. Based on the monthly climatology of Chl-a concentration in each region, three distinct winter blooming periods were considered (see Supplementary - Fig. S1): January to March for the NRS, December to February for the NCRS and SCRS, and October to January for the SRS. For the examination of the entire Red Sea basin, the analysis was based on a longer, six-month period from October to March.

### Evolution of marine heatwaves and cold-spells during winter blooming period

**Fig. 1:**
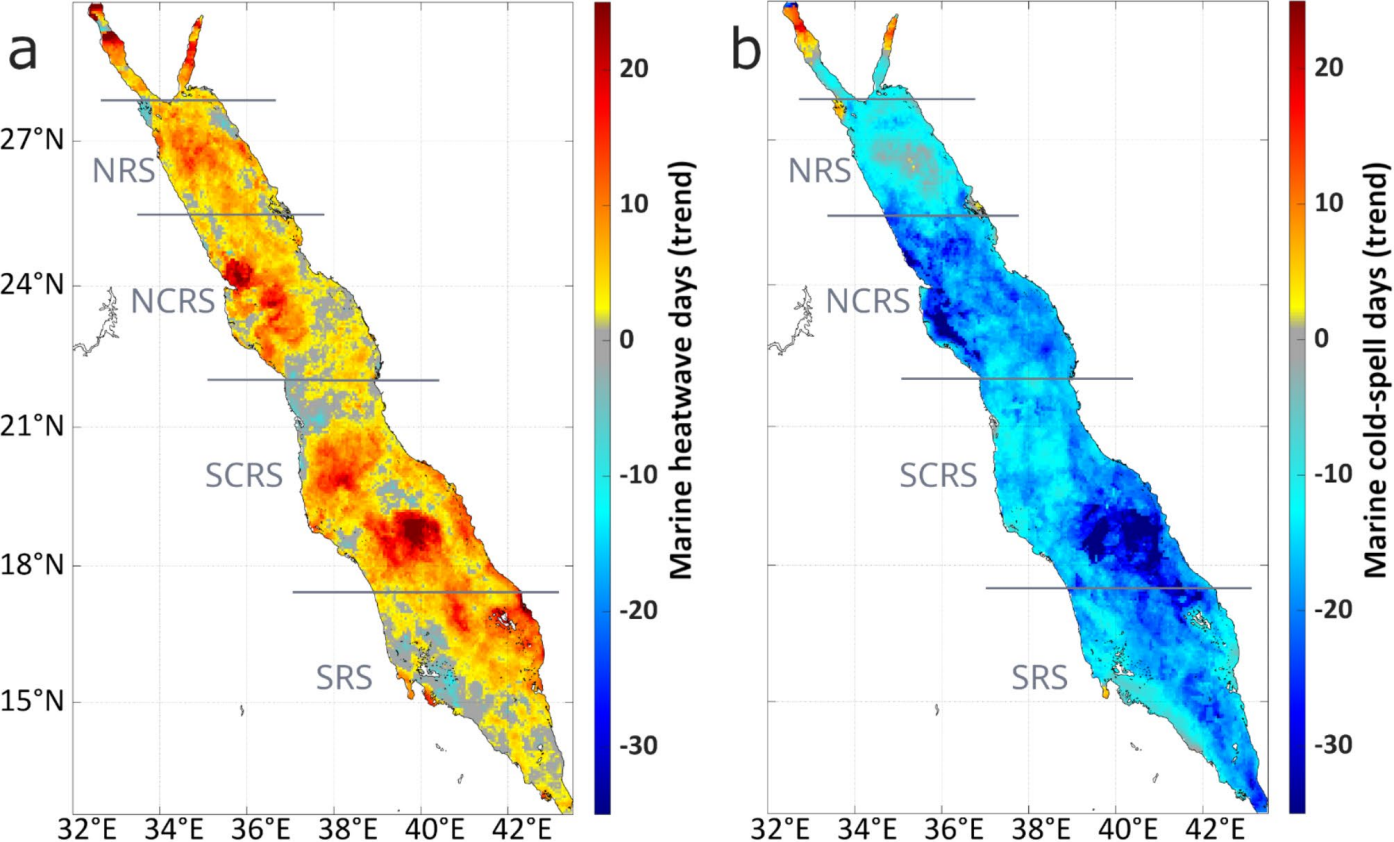
Decadal trends of the total number of MHW and MCS days during the main phytoplankton growth period (October-March) in the Red Sea, based on satellite-derived data (reprocessed OSTIA) between 1982–2018. (**a**) Trend of MHW days/decade. The horizontal grey lines divide the Red Sea into four distinct regions: Northern Red Sea (NRS), North Central Red Sea (NCRS), South Central Red Sea (SCRS), and Southern Red Sea (SRS). (**b**) Trend of MCS days/decade.

To assess the development of MHWs and MCSs and understand their influence on phytoplankton biomass we first examined their long-term trends based on the reprocessed OSTIA SST dataset (see Methods). The MHW and MCS thresholds and minimum duration were established according to Hobday et al.^6^. Between 1982 and 2018, there was a substantial increase in the total number of MHW days, for the period October to March (Fig. 1a). The decadal trend was positive throughout much of the basin, ranging from 5 to 20 additional MHW days per decade, and statistically significant in regions where higher values (> 15 days per decade) were observed (*p* < 0.05; Supplementary - Fig. S2a). The highest positive trends (> 15 days/decade) occurred in the northern areas of the Red Sea, including the Suez and Aqaba gulfs, the western part of the NCRS and the middle part of the SCRS. Although less affected, the SRS also exhibited a positive trend of 5–15 MHW days per decade (Fig. 1a).

In contrast, a decrease of approximately 10–30 MCS days per decade was observed over the entire Red Sea during the study period (Fig. 1b). This decline was particularly pronounced in the NCRS and the eastern part of the SCRS, which were characterized by at least 25 less MCS days per decade. The negative trend persisted in the NRS and SRS, but with slightly reduced magnitudes - typically fewer than 10 MCS days per decade. The central-southern part of the SRS and the areas around the Bab-el-Mandeb strait exhibited a stronger reduction of around 25 MCS days per decade or more. Overall, the decline in MCS days was statistically significant throughout the SRS, the western sectors of the central Red Sea, and the middle part of the NRS located north of the 27°N (*p* < 0.05; Supplementary - Fig. S2b).

### Phytoplankton responses to marine heatwaves and cold-spells

To assess the impact of extreme SST events (MHWs or MCSs) on Chl-a, we identified extreme Chl-a events (see methods, and^51,55,71^). When such extremes coincide, they can be referred to as compound events^72–74^. The emergence of unusual phytoplankton responses was evaluated as a result of exceptionally severe and prolonged SST extremes (see Methods for details about the conditions of Chl-a extremes and the stricter conditions of SST extremes of this section). Using a regionally-tuned ocean color dataset (based on the ESA OC-CCI product; see Methods) the Chl-a concentration (together with SST) was analyzed during the winter blooming season of each region for the 1998–2018 period, to align with the available satellite-derived Chl-a concentration data. Instead of treating each compound event as a separate case, we considered each blooming period, which included one or multiple compounds, as a single case. This approach simplifies the interpretation of results and accounts for the higher temporal variability of Chl-a concentrations compared to SST (Chl-a concentration time series are in general more “noisy”^75^).

**Fig. 2:**
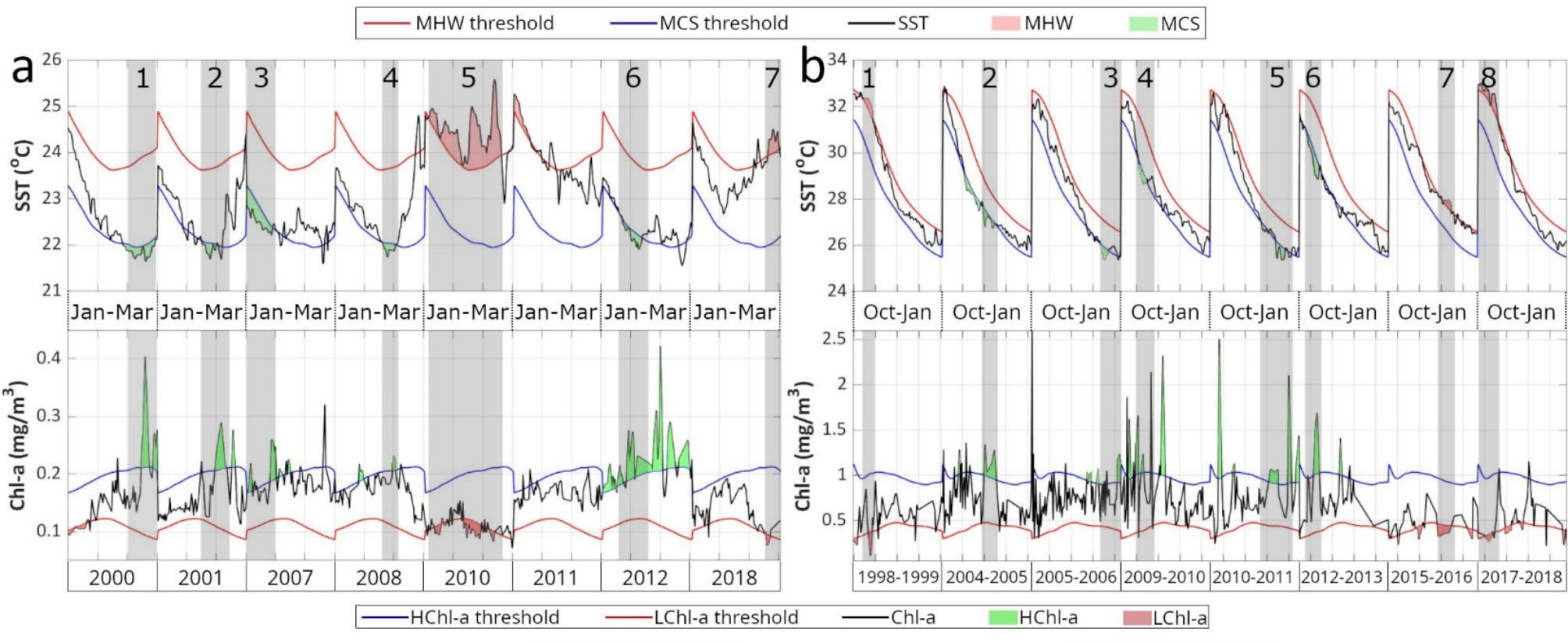
Compound events of SST – Chl-a extremes. Satellite-derived SST (C°) and Chl-a (mg/m^3^) time series, during the annual winter phytoplankton blooming periods, in the (**a**) Northern Red Sea (January to March) and (**b**) Southern Red Sea (October to January). Top plots in both panels display the SST time series: Black line represents the daily and spatially-averaged satellite SST, red (blue) line denotes the daily 92nd (8th ) percentile threshold of SST and red (green) shaded areas correspond to MHWs (MCSs). Bottom plots in both panels demonstrate time series of Chl-a concentration: Black line shows the daily and spatially-averaged satellite Chl-a concentration, blue (red) line indicates the daily 90th (10th ) percentile threshold of Chl-a and green (red) shaded areas denote HChl-a (LChl-a) extremes. The shaded grey areas (with numbers) indicate the concurrent MHW and LChl-a, or MCS and HChl-a events.

From 1998 to 2018, there were 8 annual blooming periods (January to March) in the NRS that experienced at least one extreme SST event (Fig. 2a). Notably, MHWs occurred in 2010, 2011, and 2018, while MCSs were observed in 2000, 2001, 2007, 2008, and 2012. In 7 out of these 8 cases, there was at least one concurrent Chl-a and SST extreme or a Chl-a extreme following an SST event within a maximum 1-week delay (grey shaded bars in Fig. 2a). During MHWs, the concurrent Chl-a extremes were Low Chlorophyll-a (LChl-a) events (e.g., Fig. 2a, case 5, during 2010), whereas MCSs were associated with High Chlorophyll-a (HChl-a) events (e.g., Fig. 2a, case 1, during 2000). Similarly, the SRS experienced 3 years with MHWs (1998–1999, 2015–2016, 2017–2018) and 5 years with MCSs (2004–2005, 2005–2006, 2009–2010, 2010–2011, 2012–2013) during its annual blooming period (October to January, Fig. 2b). In all 8 cases, concurrent extreme Chl-a and SST events were observed, with MHWs and MCSs consistently associated with LChl-a extremes (e.g., Fig. 2b, case 1, during 1998–1999), and HChl-a extremes (e.g., Fig. 2b, case 2, during 2004–2005), respectively.

During the 16 annual blooming periods from both regions (NRS and SRS; Fig. 2; Supplementary - Tables S1, S2) a total of 15 extreme Chl-a events were identified, where LChl-a was related to MHWs and HChl-a to MCSs. Thus, approximately 94% of the cases aligned with the expected response pattern (when each blooming period is treated as an independent case). There were no instances of unexpected Chl-a response to SST extremes (i.e., an occurrence of HChl-a during a MHW event). However, one deviation from the aforementioned pattern was noted, which occurred during the NRS MHW in January 2011, when no extreme LChl-a response was observed.

In contrast, the Chl-a response to SST extremes in the two central Red Sea regions (NCRS and SCRS) was not straightforward (Supplementary - Fig. S3; Supplementary - Tables S1, S2). Out of 13 blooming periods (December – February), a total of 11 cases were observed, (6 years in the NCRS and 5 years in the SCRS), when extreme SST events coincided with Chl-a extremes. In 3 of these cases (Supplementary - Fig. S3; cases 2 and 5 in NCRS, and case 1 in SCRS) the phytoplankton response was contrary to what was expected. Specifically, during the MHW periods of 2005–2006 and 2010–2011 in the NCRS, we observed a subsequent HChl-a instead of the expected LChl-a extreme, while the MCS of 2000–2001 in the SCRS was followed by a LChl-a event.

Most MHWs occurred in the second decade of the study period (from 2010 and onwards), whilst MCSs were mostly observed at the start of the period (see Fig. 2 and Supplementary - Fig. S3). Exceptions to this were the more recent MCS observed in 2012 (Fig. 2a, Supplementary - Fig. S3) and earlier MHW events in 2006 (Supplementary - Fig. S3a) and 1998 (Fig. 2b).

### Case studies

To investigate the physical mechanisms driving the response of phytoplankton to extreme SST events, we examined five representative case-studies of MHWs or MCSs and their impact on Chl-a concentration (mg/m^3^). Three case studies were selected, presenting the most intense MHWs or MCSs during compound events in the NRS and SRS. Additionally, two extra case-studies were chosen based on the availability of *in situ* cruise data or Argo float data.

### Phytoplankton response to specific marine heatwaves

**Fig. 3:**
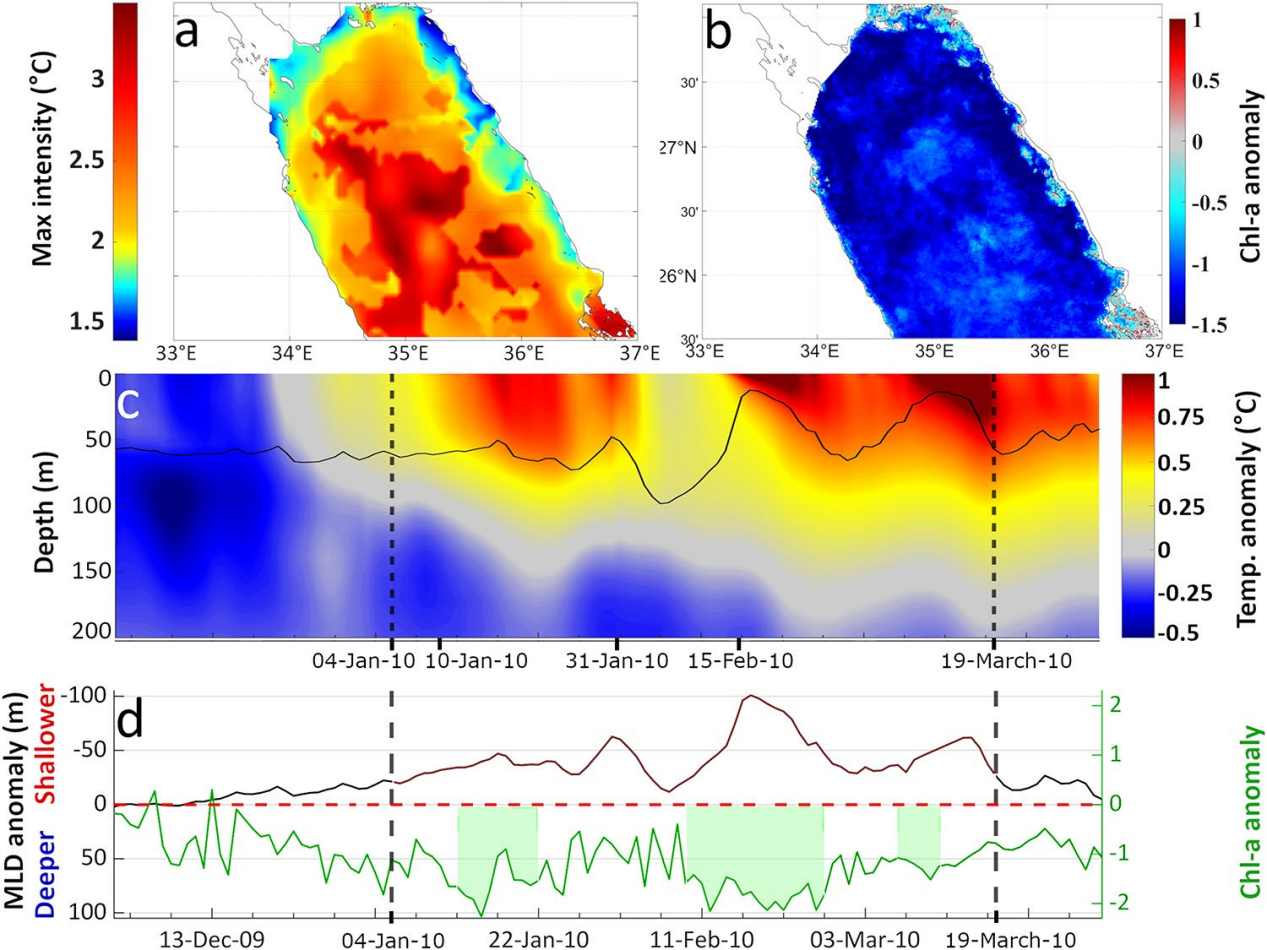
Phytoplankton response to a MHW in the Northern Red Sea (NRS), during the winter Chl-a blooming period (January to March, 2010). (**a**) Maximum intensity of the MHW. (**b**) Standardized anomaly of Chl-a concentration during one of the 3 LChl-a events (9/02/2010–26/02/2010) that coincided with the MHW. (**c**) Evolution of spatially averaged vertical temperature anomalies (°C) based on model outputs. The black line represents the spatially averaged MLD. (**d**) Spatially averaged MLD anomaly (black line – red during MHW) and standardized anomaly of Chl-a (green line); shaded green areas indicate LChl-a events. Vertical dashed lines in (c) and (d) denote the starting and ending day of the MHW.

The first case of a compound event was a prolonged (75 days; 04/01/2010 to 19/03/2010) and intense (maximum intensity of 2.7 °C) winter MHW observed in the NRS. It extended across the entire NRS, reaching its peak intensity at the center (Fig. 3a). Based on the vertical structure of temperature anomalies throughout the event (Fig. 3c), the MHW was divided into two sub-periods: the first spanning from January 10th to 31st, and a more intense second sub-period from February 15th until the termination of the event on February 26th. Both sub-periods exhibited a positive temperature anomaly of at least 0.5 °C at ~ 100 m (Fig. 3c) and a shallow MLD (~ 20 m, MLD anomaly = -100 m; Fig. 3c and d), particularly in February 2010. During the 75-day MHW, Chl-a concentration decreased considerably, with standardized anomaly values consistently below − 1.5 for the entire NRS (Fig. 3b). Multiple instances of LChl-a events coincided with this MHW (shaded green areas, Fig. 3d), which could be attributed to enhanced stratification, as indicated by the shallow MLD, associated with the abnormally warm surface and subsurface waters (Fig. 3c and d).

**Fig. 4:**
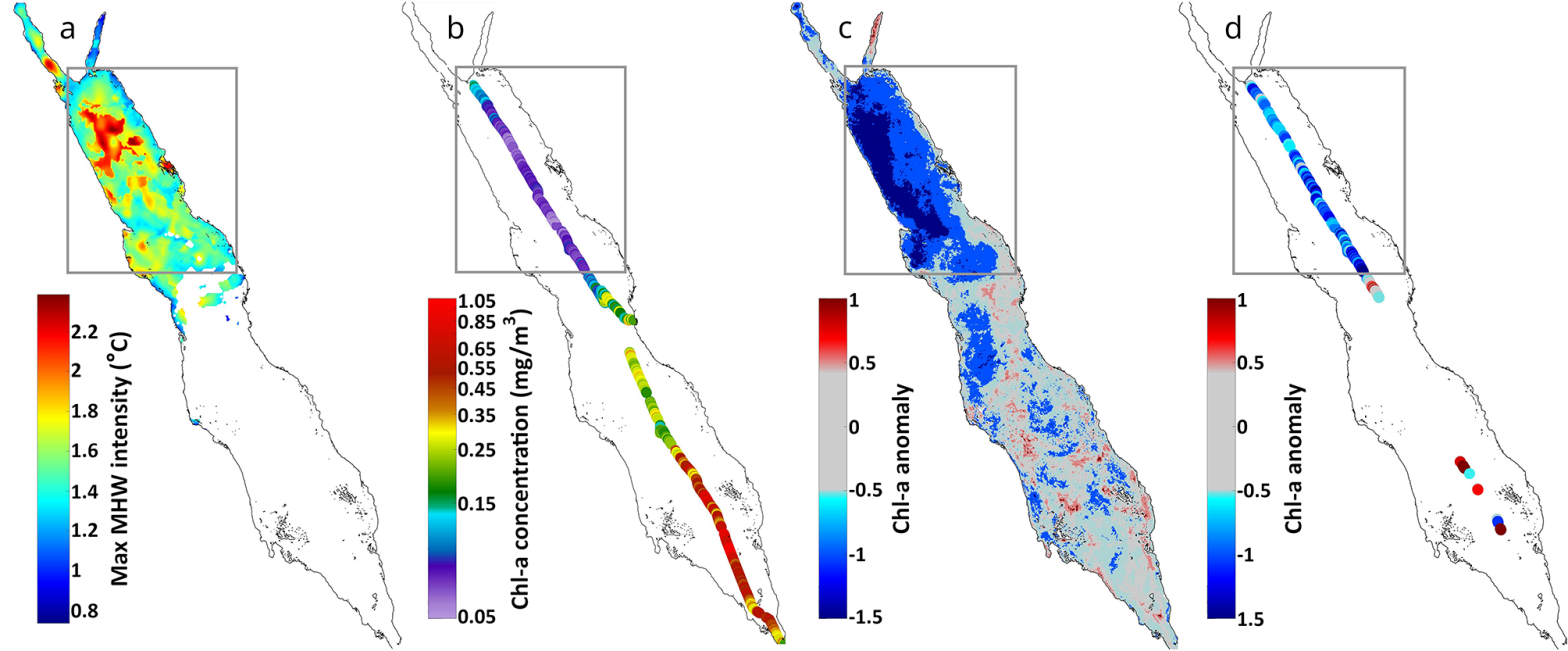
Chl-a response to a MHW event (4/01/2010–19/03/2010) in the Northern Red Sea (NRS) during the TARA research cruise in the Red Sea (08/01/2010–23/01/2010). (**a**) Maximum spatial intensity of the MHW (satellite SST data). (**b**) *In situ* Chl-a concentration values. (**c**) Standardized anomaly of satellite Chl-a concentration. (**d**) Standardized Chl-a anomaly values corresponding to the dates and coordinates of the research cruise samples (satellite matchups of the cruise samplings). The grey squares indicate the region affected by the MHW.

To further explore the impact of this strong MHW event on the spatial variability of Chl-a concentration, we used available *in situ* data collected during the Tara Oceans research cruise, which traversed the entire Red Sea within a comparable time frame (January 8th, 2010, to January 23rd, 2010; Fig. 4b; see Methods). Based on satellite-derived SST, the MHW extended to almost half of the Red Sea (Fig. 4a, grey square), with its highest intensity observed in the NRS. The *in situ* data (Fig. 4b) revealed lower Chl-a concentrations in the area affected by the MHW (ranging from approximately 0.05 to 0.1 mg/m³), compared to the higher Chl-a concentrations observed in the central (~ 0.15–0.3 mg/m³) and southern part (0.35–1 mg/m³) of the Red Sea.

The Red Sea is characterized by higher Chl-a concentrations towards the south^70^, and thus may not be necessarily linked to this MHW event. However, an analysis of Chl-a concentration anomalies, based on 21 years of satellite data (1998–2018), suggests that Chl-a concentrations were indeed anomalously lower, relative to the climatological mean (Fig. 4c and d). The standardized anomalies of Chl-a concentration revealed a decrease of 0.5 to 1.5 standard deviations over the region affected by the MHW, whilst the remainder of the Red Sea exhibited Chl-a values close to climatology or higher. These deviations were evident in both the Chl-a anomalies observed from the satellite (averaged over the cruise period; Fig. 4c) and those derived from satellite data matching the specific dates and coordinates of the research cruise samples (Fig. 4d; satellite matchups to *in situ* sampling).

**Fig. 5:**
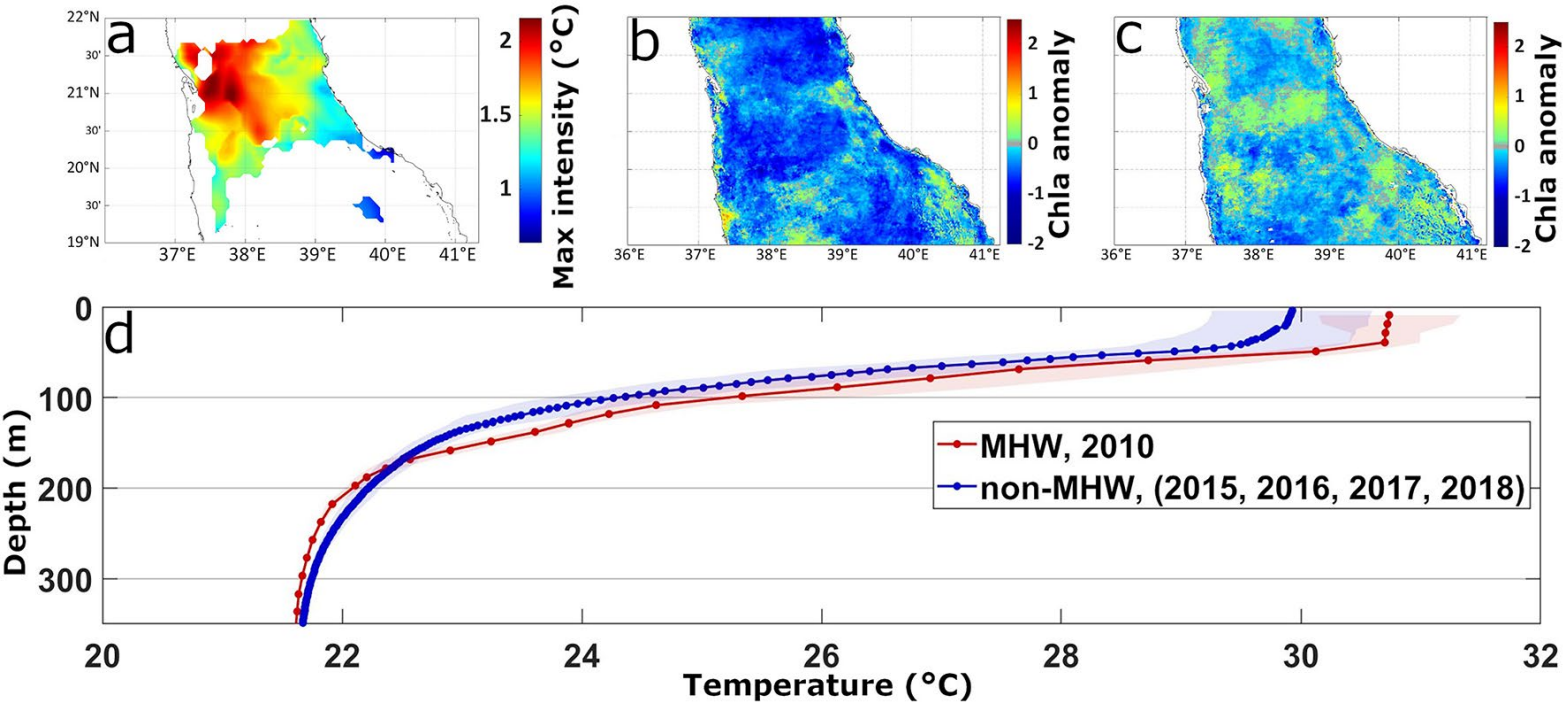
Characteristics of the MHW and phytoplankton response between 7/11/2010–3/12/2010 in the South Central Red Sea (SCRS). (**a**) Spatial variability of maximum MHW intensity based on satellite SST. The standardized anomalies of satellite Chl-a are indicated for the (**b**) the MHW and (**c**) non-MHW years (2015–2018). (**d**) Comparison of the spatially-averaged vertical temperature profile during MHW days (red line) with the equivalent (7/11 − 3/12) mean temperature profile of non-MHW years (blue line).

Moving southwards, the available Argo float data were used (see Methods) to examine an *in situ* case study of a MHW in the SCRS (7/11/2010–3/12/2010, Fig. 5a, S4a - red circles) and compare subsurface conditions between a MHW (2010) and non-MHW years (2015–2018) (Supplementary - Fig. S4). This MHW predominantly impacted waters between 20° − 22°N, with maximum intensities exceeding 2 °C (Fig. 5a). Argo temperature profiles revealed a well-defined, stratified surface layer down to ~ 50 m depth with temperatures reaching ~ 31 °C during the MHW (red line, Fig. 5d). Beyond this layer, temperatures rapidly declined to ~ 25 °C at 100 m depth. In contrast, the equivalent mean vertical temperature profile during non-MHW years showed a smoother transition of temperatures from the surface layer to the thermocline, with temperatures remaining below 30 °C (Fig. 5d blue line, Supplementary - Fig. S4a). This indicates a higher degree of mixing during non-MHW years, compared to the observed MHW conditions. Phytoplankton biomass within the MHW area, characterized by higher SST intensities (1.5–2^o^C), experienced a substantial reduction in late 2010 relative to non-MHW years (Fig. 5b, Supplementary - Figs. S4a, b), with Chl-a values reaching 2 standard deviations below the mean. In contrast, Chl-a concentrations during non-MHW periods remained closer to, or higher than, climatological values between 20^o^ and 21^o^N (Fig. 5c, Supplementary - Fig. S4c).

### Phytoplankton response to specific marine cold-spells

**Fig. 6:**
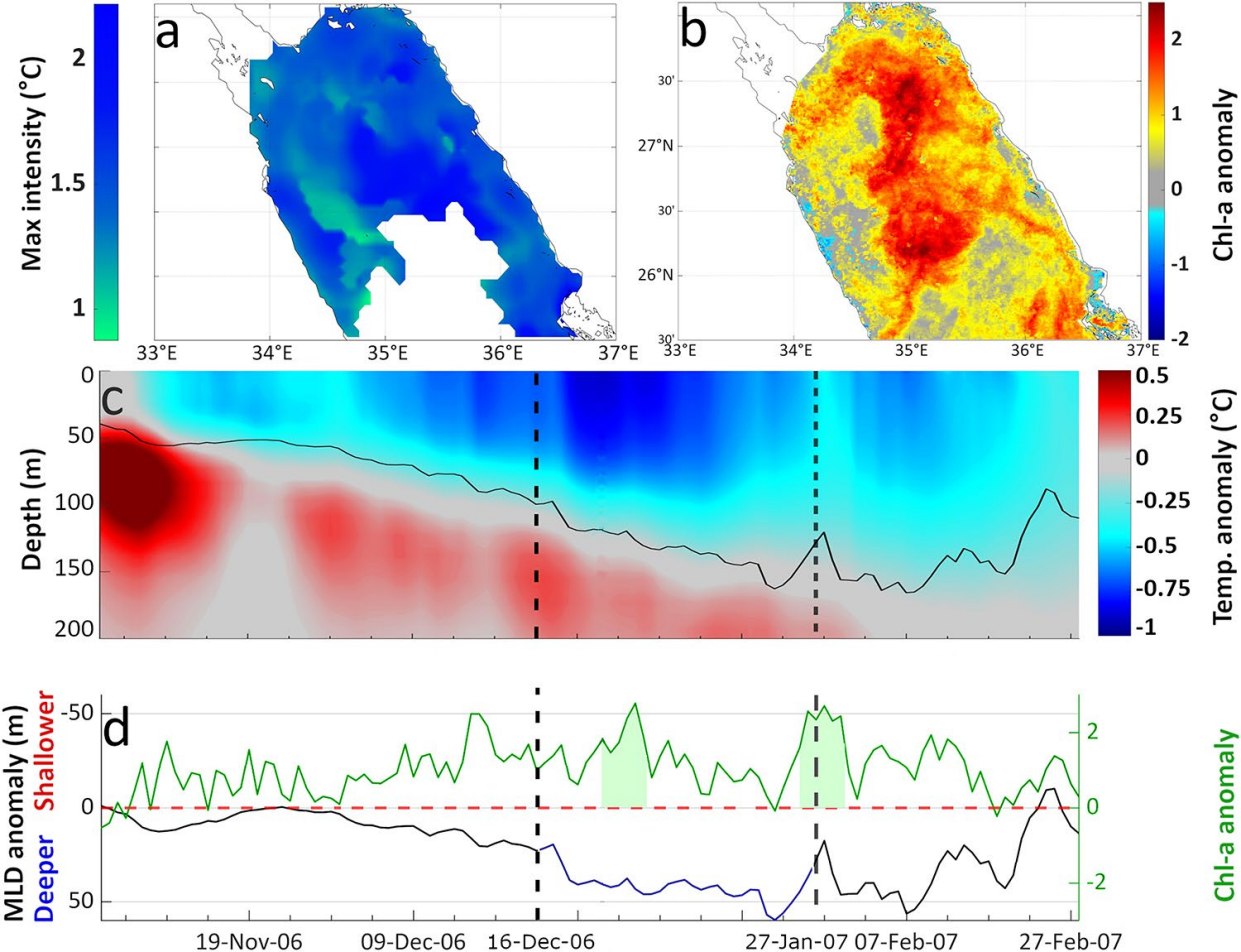
Phytoplankton response to the MCS between 16/12/2006–27/01/2007 in the Northern Red Sea (NRS), during winter Chl-a blooming period (January to March). (**a**) Spatial variability of maximum MCS intensity based on satellite SST. (**b** )Standardized anomaly of Chl-a concentration during the longest HChl-a event (30/12/2006–6/1/2007) coinciding with the start of the MCS. (**c**) Evolution of the spatially-averaged profile of vertical temperature anomaly (°C) and MLD based on model outputs (see Methods); vertical dashed lines indicate the starting and ending day of the MCS. (**d**) Spatially averaged MLD anomaly from model data (black line) and standardized anomalies of satellite-Chl-a (green line); shaded green areas represent the detected HChl-a events.

The longest (45 days) and most intense (averaged maximum intensity of -1.4 °C) MCS was observed in the NRS (Fig. 2a, case 3) between 14/12/2006–27/1/2007. This winter event covered the majority of the NRS (Fig. 6a), with maximum intensity exceeding 2 °C in the central NRS. During the MCS, temperature anomalies reached − 1 °C at 50 m and − 0.5 °C at 100 m (Fig. 6c), whilst the MLD ranged from 100 m to 150 m (Fig. 6c). Together with the anomalously deep MLD (50 m deeper than the climatology of 2001–2015, Fig. 6d), colder surface and subsurface temperatures indicate the presence of nutrient-rich waters originating from the deeper layers of the water column during this event.

In addition, Chl-a anomalies approached or exceeded 2 standard deviations from the climatological mean throughout the entirety of the event (Fig. 6d). This increase in phytoplankton biomass was further highlighted by the occurrence of two HChl-a extremes at the start (30/12/2006–6/1/2007) and towards the end (25/1/2007–30/1/2007) of the MCS (Fig. 6d). The first Chl-a event – lasting 8 days – occurred over the entire NRS, with the highest Chl-a anomalies ( > = 2) observed in the center of the NRS.

**Fig. 7:**
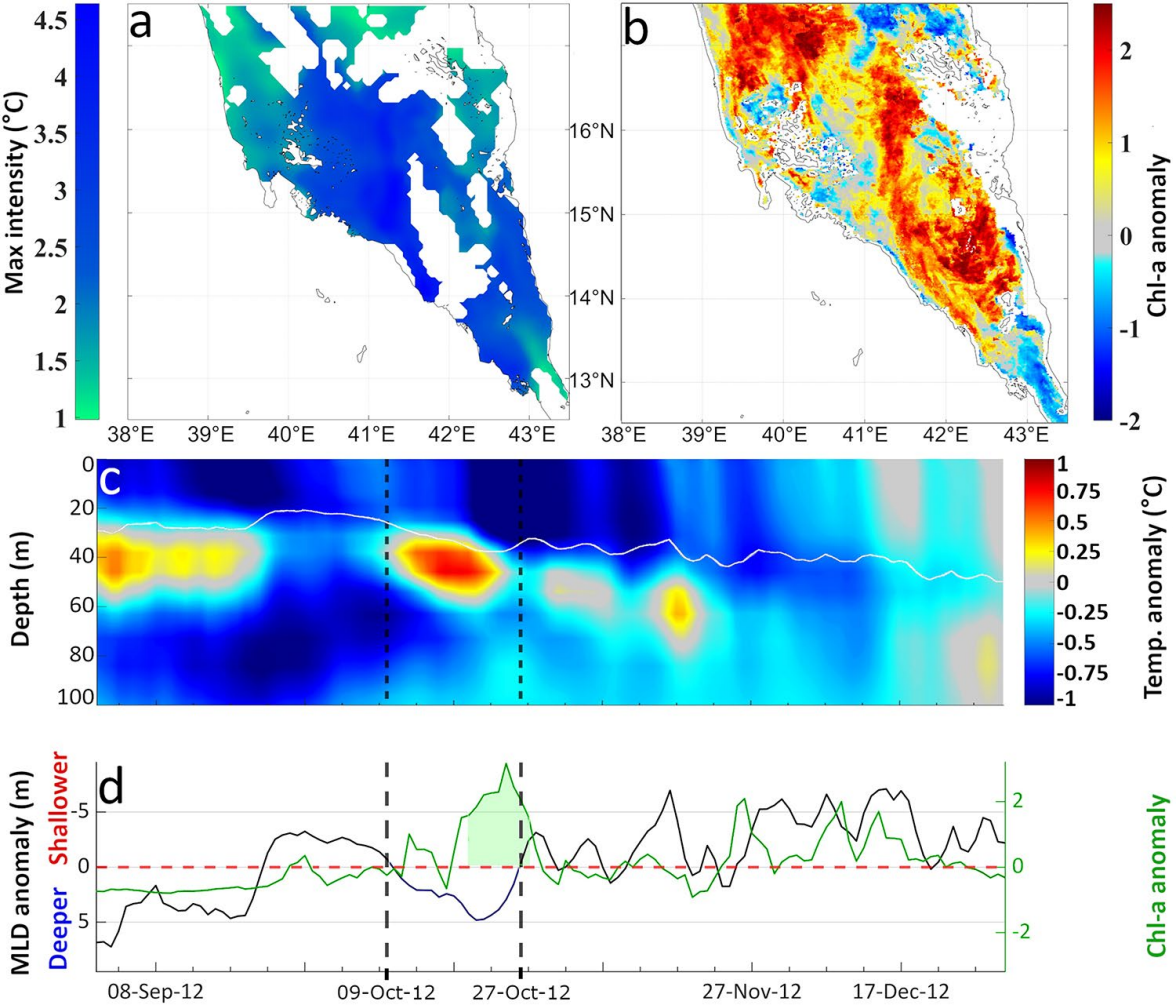
Phytoplankton response to a MCS event (9/10/2012–27/10/2012) in the Southern Red Sea (SRS), during the winter blooming period (October to January). (**a**) Spatial variability of maximum MCS intensity. (**b**) Standardized anomaly of Chl-a concentration during the HChl-a event (20/10/2012–28/10/2012) coinciding with the MCS. (**c**) Model output of the spatially-averaged, vertical profiles of temperature anomaly (°C) and MLD (m); vertical dashed lines indicate the MCS period. (**d**) Model output of the spatially-averaged MLD anomaly (black line) and standardized anomaly of the satellite Chl-a (green line); shaded green area represents the detected HChl-a event.

In the SRS, the longest (19 days) and most intense (maximum intensity of -1.9 °C) MCS that coincided with a HChl-a extreme occurred between 9/10/2012–27/10/2012 (Fig. 2b, case 6; Fig. 7a). This event covered almost the entire SRS (Fig. 7a), reaching its peak intensity (> 4 °C) in the central, deeper parts of the region. Throughout the event’s duration, a negative temperature anomaly of ~ 1 °C was observed from the surface to approximately 40 m, corresponding to the average MLD of the SRS (Fig. 7c). The MLD was about 5 m deeper than normal (Fig. 7d), extending down to the layer of nutrient-rich Gulf of Aden Intermediate Water (GAIW) (Fig. 7c, Supplementary - Fig. S5;^76^). Chl-a concentrations showed a gradual increase (Fig. 7a; Chl-a standardized anomaly = 2), leading to a HChl-a event from 20/10/2012–28/10/2012 (Fig. 7d). The peak Chl-a values of this event were observed mostly in the open, deeper waters of the SRS (Fig. 7b).

## Discussion

Global warming is anticipated to impact the rate of occurrence and intensity of extreme temperature events (MHWs and MCSs), thereby influencing marine ecosystems to varying degrees^5,7,8^. Despite increasing efforts to comprehend the response of marine ecosystems to such extreme temperature events, the available information remains limited. One of the challenges associated with these extreme cases is the abruptness of their occurrence, ultimately altering the capacity of organisms or ecosystems to adapt accordingly^31^. In this study, a rise in MHWs and decline in MCSs were observed over the Red Sea, during the winter blooming periods, over a 37-year period (1982–2018). These trends have substantial implications for phytoplankton biomass, especially in the northern and southern regions of the basin. We identified distinct phytoplankton responses to MHWs and MCSs, characterized by extremes of HChl-a and LChl-a concentration, respectively. The analysis of specific case studies shows that anomalous deepening of MLD occurred during compound MCS-HChl-a events, whereas anomalously high surface and subsurface temperatures coincided with a shallower MLD during MHW-LChl-a events.

Our findings revealed a significant rise in MHW occurrence across the Red Sea, which aligns with the rapidly warming trend of the basin^25–27^ and the global increase in MHW frequency, intensity, and duration^77,78^. It is worth noting that the increase in MHW days was observed during the winter blooming period and was more pronounced (> 15 days/decade, years 1982–2018) in the central NRS, the western part of the NCRS and the mid-south part of the SCRS, where eddy activity is consistently present^70,79,80^. The most substantial increases in MHW days were observed in the gulfs of Suez and Aqaba, where MHWs exceeded twenty days per decade (years 1982–2018). However, the trend of MHWs in the two gulfs is not statistically significant (Supplementary - Fig. S2; p-value > 0.05), so we should be cautious in interpreting this result, until new data from the coming years are included. This is especially important since the northern parts of the Suez and Aqaba gulfs also exhibited a statistically significant (Supplementary - Fig. S2; p-value < 0.05) increasing trend in MCS days. Given that MCSs can be associated with deeper MLDs, this result may reflect a known tendency for deeper MLDs in the northern gulf of Aqaba during winter, in years of deep mixing^81–85^. In addition, vertically-integrated phytoplankton growth has been shown to increase because of enhanced horizontal advection, during MLD deepening in that region^84^. Hence, future studies focusing on the spatial variability of MLD and phytoplankton dynamics in the gulf of Aqaba could benefit from examining the evolution of MHWs and MCSs.

In contrast to the gulfs of Aqaba and Suez, there has been a substantial decline in the observed number of winter MCS days over the entire Red Sea. This decline aligns with the documented declining trend of global MCSs^5^. This decreasing MCS trend, generally amounting to 10–30 MCS days less per decade, was particularly evident (at least 25 fewer MCS days per decade) in the western NCRS, the eastern SCRS, and in parts of the SRS. The reduction in MCS days may be influenced by the general warming of the basin^25–27^. This warming has been attributed to reduced winter atmospheric cooling^86^, while particularly in the southern part of the basin, may be influenced by the intrusion of warm surface waters from the West Indian Ocean (WIO) during winter, through the Bab-el-Mandeb strait^87,88^. This intrusion is more pronounced during positive phases of the Multivariate El Niño–Southern Oscillation Index^36^, extreme cases of which are anticipated to become more frequent^89^. Furthermore, the infiltrating surface waters are getting warmer due to the intensified warming of the WIO^90,91^, while the heat transfer towards the broader region (e.g., Arabian Sea, Gulf of Aden) is expected to be further enhanced^92^. Overall, the decline in MCS days generally accelerated more rapidly compared to the increase in MHW days, since the negative trend of MCS across the basin was 10–30 days/decade, in contrast to the positive MHW trend of 5–20 days/decade. In addition to the increasing (decreasing) trend in MHW (MCS) days per decade, most MHWs occurred after 2010, in contrast to MCSs that were primarily observed before that year (see Fig. 2, S3). Both shifts are indicative of consistently warmer temperatures over the recent decade.

Drawing on general knowledge of the productivity in tropical marine ecosystems^43^, phytoplankton could potentially benefit from MCSs and the accompanying well-mixed, nutrient-rich surface water. Since phytoplankton constitute the base of marine food webs, alterations to phytoplankton abundance and the timing of their growth (phenology), can reverberate through marine ecosystems, as the survival and fitness of organisms at higher levels of the food web is dependent on food availability^93,94^. Additionally, it may enhance carbon transport to deeper waters due to its role in the biological carbon pump^38,39,68^. Conversely, phytoplankton biomass could be negatively impacted during MHWs, which are associated with increased stratification and reduced nutrient availability. This scenario may negatively affect higher trophic level populations and decrease the ocean’s absorption of atmospheric CO_2_. Such an approach is primarily applicable to open waters at low and mid latitudes, where the main source of nutrients originates from deeper water^9,49^. However, it may not always apply to coastal waters of marginal seas, where upwelling mechanisms are subject to different drivers of variability and trends, depending on the region and climate change scenario^95–97^. The open-water hypothesis was confirmed for the NRS and SRS (Fig. 2). Phytoplankton biomass in the Red Sea depends on the typical mechanism of vertical-mixing during winter, the influx of water masses from the Indian Ocean^76,98^, and the extended (> 5000 km) nutrient-rich coral reef ecosystems that dominate most of the coastal zone^99,100^. The NRS is described as a typical tropical oligotrophic marine ecosystem^35^, where nutrient redistribution from vertical mixing has a key role in fertilizing the upper layers of the ocean, resulting in winter phytoplankton blooms^35,36,101^. Hence, the occurrence of warmer, more stratified conditions can lead to a decline in phytoplankton abundance there. Our findings for the NRS are consistent with this mechanism, revealing LChl-a extremes during MHWs and HChl-a during MCSs (Figs. 2a, 3, 4 and 6). In the SRS, nutrient influx into the basin primarily occurs through the narrow Bab-el-Mandeb strait^36,87^. During winter, northward-blowing winds facilitate the intrusion of nutrient-rich surface water from the Gulf of Aden into the basin (inverse estuarine circulation), directly influencing the entire Red Sea except for the NRS^36^. During the summer, however, the strait circulation reverses, creating estuarine conditions characterized by an outflow of Red Sea surface water^76,98^ and an influx of nutrient-rich Gulf of Aden Intermediate water^76^. The subsurface GAIW remains in the SRS until the start of the winter blooming period (October to January), occupying depths between 40 m and 70 m. GAIW is characterized by lower salinities (typically between 36.5 and 37.5 psu) and cooler temperatures (as low as 20 °C) than surface waters^87,88,102^. In our case-study, a temperature anomaly at around 40 m was observed during a compound MCS-HChl-a event in the SRS (Fig. 7c), which was likely related to the GAIW, as also indicated by the layer’s temperature and salinity (Supplementary - Fig. S5). We provide evidence (Fig. 7) that during winter MCSs in the SRS, the MLD extends down to the zone of GAIW influence (Fig. 7c), resulting in a distinctive phytoplankton bloom (Fig. 7b and d), indicating potential fertilization of the upper ocean layers. In agreement with Dreano et al.^87^, we highlight the significance of GAIW in promoting regional phytoplankton blooms in the SRS through local mixing and upwelling (Figs. 2b and 7).

Within the NRS and SRS, a total of sixteen annual blooming periods were reported, where MHWs and MCSs occurred, between 1998 and 2018. In 15 of these instances, constituting approximately 94% of them, there was a concurrent LChl-a event in response to MHWs and a HChl-a event in response to MCSs. Notably, no cases of an opposite phytoplankton response were observed, (such as HChl-a during MHWs) in these areas. Our results align with prior research that has reported reduced Chl-a concentrations during MHW events in nutrient-limited regions at low and mid latitudes^9,49,51^, as well as increased Chl-a concentrations during MCS events^5,62,103^. It has been previously suggested that there exists a systematic relationship, rather than random co-occurrence, between SST and Chl-a extremes^9,49,55,59^. In our detailed analysis of specific case-studies (Figs. 3, 6 and 7), it was further confirmed that combined MHW-LChl-a extremes are associated with unusually shallow MLD and stratified water conditions, while MCS-HChl-a extremes are linked to deep MLD and well-mixed surface waters. Hence, it is proposed that the interplay between MHWs and shallower MLD leads to LChl-a extremes, whereas the MCSs and deeper MLD result in HChl-a events, due to the increased nutrient availability. However, nutrient availability is not exclusively linked to the described mechanism for all regions of the Red Sea. Our study underscores such a distinction, as straightforward Chl-a responses during MHWs and MCSs in the central part of the basin were not consistently observed.

Interestingly, the described MHW – LChl-a and MCS – HChl-a coupling was less obvious in the NCRS and SCRS provinces (Supplementary - Fig. S3). There, 3 out of the 11 compound events identified, demonstrated the opposite phytoplankton response: two cases exhibited increased Chl-a concentrations during MHWs, and one displayed a Chl-a decrease during MCSs. The observed disparity may be attributed to variations in nutrient availability mechanisms. The central Red Sea is characterized by a more complex hydrodynamic environment compared to the rest of the basin. It encompasses eddies that are not only frequent, but also large with respect to their mean zonal radius, persisting for long time periods (6 to 9 weeks^104,105^). Such highly dynamic circulation patterns^76,98,104,106,107^ have been shown to play a significant role in horizontally-transporting water masses from coastal and coral reef regions to offshore waters^99^. These transported waters can carry additional nutrients, detritus, and phytoplankton populations. Furthermore, these eddies can alter vertical water column features, like uplifting isopycnals and inducing vertical mixing (e.g., in their periphery) unrelated to surface cooling convection. Overall, the central Red Sea presents a notably dynamic system with multiple nutrient sources, not limited to convective mixing by surface cooling. This multifaceted nutrient supply could account for the deviation of phytoplankton responses from our hypothesis in this area.

Phytoplankton responses to SST extremes were assessed and quantified utilizing the concept of Chl-a extremes. This approach drew upon the methodology initially proposed for SST extremes by Hobday et al.^6^ and has been previously employed for various ecosystem variables, including acidification, oxygen levels, and Chl-a concentration, to identify both compound^55,74^ and individual extreme events for each variable^71^. We established the intensity and duration thresholds for MHWs (MCSs), and HChl-a (LChl-a) events based on a balanced ratio. These thresholds were chosen to ensure that temperature extremes were severe and long enough to investigate their impacts on phytoplankton biomass, while still allowing for an adequate number of compound events. These thresholds are recommended for future studies with similar objectives in the Red Sea. However, these criteria should be tailored to the specific region and scientific inquiry^6,8^. For example, Genevier et al.^19^ employed a 95th percentile temperature threshold and a 7-day minimum duration, to identify MHWs related to major coral bleaching in the Red Sea.

## Conclusion

The substantially decreased Chl-a concentration is linked to MHWs and a shallower MLD, whereas higher phytoplankton biomass is related to MCSs and a deeper MLD in the Red Sea, especially in regions where surface cooling drives convective mixing (deeper MLD), promoting nutrient availability. MHWs and MCSs appear to evolve in opposite directions, with an increase in MHW days per decade and a decrease in MCS days.

Our study underscores the negative effects of combined increasing MHWs and declining MCSs trends on phytoplankton biomass in the Red Sea. Given that the MCSs are generally associated with deeper vertical mixing, leading to higher nutrient availability, the significant decrease in winter MCS events in the Red Sea may be more crucial for phytoplankton growth compared to the gradual increase in MHW events. Should these trends persist, as anticipated at both global^24,108,109^ and regional scales^24,25^, the adverse responses of phytoplankton may potentially intensify in the coming years. Given the significance of phytoplankton for marine ecosystems, either as a foundational component supporting the entire trophic web^37^ or as a key player in sequestering substantial quantities of atmospheric CO_2_^38,39,68^, such a scenario could trigger a cascade of detrimental consequences for marine ecosystems.

The Red Sea, with its extensive endemic species, rich biodiversity^28^, and its provision of vital economic services to the broader region including tourism (e.g. NEOM coastal city megaproject^110^), shipping, and fisheries^29,111,112^, warrants particular attention in the context of climate change. This concern also extends to other marginal semi-enclosed seas similarly susceptible to ocean warming^24^.

Future research on phytoplankton responses to SST extremes in nutrient-limited open-water marine ecosystems at mid and low latitudes could benefit from integrating MLD responses to MHWs/MCSs and Low Chlorophyll-a/High Chlorophyll-a extremes. Furthermore, understanding the potential driving mechanisms behind such extreme phenomena^9,113–115^ will offer valuable insights into the future of our oceans under various warming scenarios. Methodological approaches that combine a suite of oceanographic tools and datasets to provide comprehensive information on the surface and subsurface oceanic conditions, while specifying extreme Chl-a events in relation to corresponding SST extremes, will offer a better understanding of marine-life responses to a warmer world.

## Methods

### Satellite derived sea surface temperature data

To analyze extreme temperatures in the Red Sea, daily Sea Surface Temperature (SST) satellite data were used, between 1982 and 2018. SST data were retrieved from the “Global Ocean Operational SST and Sea Ice Analysis (OSTIA) Sea Surface Temperature and Sea Ice Reprocessed” product (10.48670/moi-00168*)*, of the E.U. Copernicus Marine Environment Monitoring Service (CMEMS) ocean data portal. OSTIA utilizes a combination of satellite information gathered from microwave and infrared satellite instruments, delivered by the Group for High Resolution SST (GHRSST), in conjunction with *in situ* observations sourced from the International Comprehensive Ocean-Atmosphere Data Set (ICOADS) database. The current product is a level 4, daily, global sea surface temperature reprocessed product with a horizontal resolution of 0.05°x 0.05°. It was produced using *in situ* and satellite data^116^, initially provided by the UK Met Office. Validation of the SST product using drifting buoy data for the entire period and near-surface (3–5 m) Argo data for the most recent 16 years consistently demonstrated a close alignment between the analysis and the observed data. Further detailed information are available in the “Quality Information Document” (https://catalogue.marine.copernicus.eu/documents/QUID/CMEMS-SST-QUID-010-011.pdf*).* Satellite-derived data were further spatially-averaged to produce daily SST time series.

### Satellite ocean color data

 Daily Chl-a data were acquired from a dataset specifically produced and regionally tuned to the Red Sea, at a 1 km spatial resolution, between 1998 and 2018, using a regional ocean colour algorithm described by Brewin et al.^117^. The Chl-a dataset is an output of the European Space Agency’s Ocean Colour Climate Change Initiative (ESA OC-CCI, Version 3.1) and an additional data processing through the United Kingdom Natural Environment Research Council—Earth Observation Data Acquisition and Analysis Service (NEODAAS). In general, it has been shown that both standard ocean-color algorithms and the regionally-tuned OC-CCI algorithm used in this study, perform well in the Red Sea, arguing in favor of the use of satellite-derived datasets^66,117–120^. The OC-CCI product provides a significantly higher amount of data, compared to single-sensor-based datasets^36^. The regionally tuned Chl-a dataset used in our study has been proved to perform significantly better than other standard and semi-analytical datasets^117^, and it is – to our knowledge – the highest quality product available for the Red Sea. For more information, we refer the reader to previous studies where this specific dataset was used^110,117,120^, as well as to the OC-CCI product user guide (http://www.esa-oceancolour-cci.org/?q=webfm_send/318*).* To construct dependable daily and monthly climatologies of Chl-a concentration, daily satellite data with a spatial coverage less than 30% were removed, together with grid points of Chl-a concentration exceeding 10 mg/m^3^, as they were considered as false, extreme values for the area. Missing values present in spatially-averaged daily time series were filled with linear interpolation and daily anomalies of Chl-a concentration were computed relative to the climatological Chl-a mean, of 1998–2018. We also computed standardized Chl-a anomalies [(specific variable value - climatological value)/standard deviation], with standard deviation referring to the temporal standard deviation, in relation to the climatological values.

### *In situ* based chlorophyll-a data

Measurements of Chl-a concentration (mg/m^3^) were collected during the Tara Oceans expedition (Tara) that took place in the Red Sea during January 2010. The data were collected on a flow-through system using a WET Labs AC-S hyper-spectral spectrophotometer and Sea-Bird Electronics SBE45 MicroTSG unit^121–123^. They were obtained from the NASA SeaBASS website (http://seabass.gsfc.nasa.gov/^124^) and were processed by Brewin et al.^117^ according to the methods described by Slade et al.^125^. For more detailed information on the measurement and processing of particulate absorption and attenuation, as well as the Chl-a concentration during Tara expedition, readers are referred to Boss et al.^121^ and Werdell et al.^123^.

### Argo-float data

The selection and analysis of the case study presented in Fig. 5 and S4, was determined by the availability of Argo-floats data in the basin, based on specific criteria, i.e., at least one Argo-float trajectory had to coincide with an SST extreme event within a particular region, while several other Argo-floats needed to traverse the same region during years devoid of extreme events. These conditions were essential to create suitable reference data for a comparative analysis. Only a single Argo-float trajectory was found to satisfy these criteria, covering a 27-day-long MHW between 7/11/2010–3/12/2010 in the central Red Sea (Fig. 5a, S4A - red circles). To compare with the subsurface conditions of the equivalent period during years without extreme events (2015–2018) we used data from two additional Argo-floats (see Supplementary - Fig. S4).

Core-Argo float data were retrieved from the online data selection tool of the Euro-Argo European Research Infrastructure Consortium (ERIC) (https://dataselection.euro-argo.eu/). These data were collected and made freely available by the International Argo Program and the national programs that contribute to it (https://argo.ucsd.edu ,https://www.ocean-ops.org ). The Argo Program is part of the Global Ocean Observing System (Argo, 2000). The three Core-Argo floats that were acquired [ (1) WMO ID: 2901098; https://www.ocean-ops.org/board/wa/InspectPtfModule?ref=2901098, (2) WMO ID: 1900959; https://www.ocean-ops.org/board/wa/InspectPtfModule?ref=1900959, *(3)* WMO ID: 1900960; https://www.ocean-ops.org/board/wa/InspectPtfModule?ref=1900960*]* provide information on the physical characteristics of the water column during 2010, and between 2015 and 2018. The first float (#2901098) had a cycle time of approximately 5 days, drifting depth at 400 m, and maximum profile depth at 600 m. The remaining two Argo-floats had cycle times of 4 days, drifting at 1000 m, and profile depth of 1500 m. They all bore SEABIRD_SBE41 sensors for measuring salinity, temperature, and pressure, while the first Argo-float was also equipped with an extra sensor (KISTLER_2900PSIA) for pressure. We used the ascending profiles’ adjusted values for temperature, salinity, and pressure of “good quality” data (flag value = 1). Using the *in situ* temperatures, we further calculated the Mixed Layer Depth (MLD) based on temperature-difference criterion of 0.2 C^o^ between the surface layer (10 m) and the deeper water layers, using the CSIRO Seawater EOS80 package (CSIRO, 2014; http://www.cmar.csiro.au/datacentre/ext_docs/seawater.html*).*

### Model derived 3D temperature data

 To examine the variability and climatological profiles of temperature in the water column, we obtained 3D temperature outputs from a high resolution (~ 1 km and 50 vertical layers) regional hydrodynamic simulation of the Red Sea and the adjacent gulfs over the period 2001–2015. of the Massachusetts Institute of Technology general circulation model (MITgcm)^126,127^. The model was forced by a downscaled regional atmospheric reanalysis covering the Red Sea and the neighbouring regions with a spatial resolution of ~ 5 km^69,128,129^. It has been previously used to study basin-scale circulation dynamics and mesoscale eddy activity in the Red Sea^76,80,104,105,130^, the water exchanges with the open ocean^88^, as well as the biophysical connectivity in the basin^131^. Of special importance for this study, the current version of the model setup has been extensively validated against available observations, focusing primarily on the upper layer properties and mixed layer dynamics^127^. Specifically, the model results have been evaluated against historical CTD observations covering the entire simulation period and throughout the Red Sea, as well as satellite sea surface temperature data. Daily outputs of 3D temperature fields were used to estimate the mixed layer depth, create climatological profiles, and examine the variability of temperature in the water column.

### Extreme event detection

#### Marine heatwaves/marine cold-spells

MHWs (MCSs) have been suggested to occur when seawater temperatures exceed (are lower than) the climatological 90th (10th) percentile of temperature, for at least five consecutive days (MHW;^6^) (MCS;^5^). In the first part of the study, we examine the long-term trends of MHWs and MCSs with these characteristics between 1982 and 2018 over the entire Red Sea (Results; Sect. 2.1). We assess their linear trends using linear least squares regression (Fig. 1) and their statistical significance using the p-value of the two-sided t-statistic test, at a 0.05 significance level (Supplementary - Fig. S2), from the MATLAB toolbox M_MHW^132^. Given that the nature of detected extreme temperature events can vary based on the specific scientific question and the region in focus, it is recommended to adjust thresholds and minimum event durations to align with the desired characteristics^6,8,50^. Therefore, for the second part of the study (Results; Sect. 2.2), MHWs (MCSs) were detected when temperature exceeded (was lower than) the climatological 92nd (8th) percentile and a minimum duration of ten days. These thresholds for the intensity and duration of MHWs and MCSs were selected to focus on the longest and strongest SST extremes that cause clear and measurable – in the form of Chl-a extremes – phytoplankton responses (see also next section of Methods). To be consistent with the available Chl-a concentration data, the temperature reference period (against which MHWs are identified) for the second part of the study was limited to 1998–2018. In both sections, we applied the respective MHW and MCS detection algorithms, proposed by Hobday et al.^6^ and Schlegel et al.^5^, on the satellite SST dataset (reprocessed OSTIA) to identify events and compute their duration, intensity, frequency, start and end (in days). The computations were performed with the MATLAB toolbox M_MHW^132^; toolbox available at https://github.com/ZijieZhaoMMHW/m_mhw1.0*).* The temperature climatology was computed following Hobday et al.^6^, whereby the mean is calculated within an 11-day window centred around each “climatological” day and further smoothing is applied with a 30-day moving window mean.

### Chlorophyll-a extreme events

To identify events of anomalously High Chl-a (HChl-a) and Low Chl-a (LChl-a) concentration we applied the MHW/MCS algorithm^5,6^ on the available Chl-a data. We set HChl-a (LChl-a) extremes as the events where Chl-a values exceeded (were lower than) the climatology of the 90th (10th ) percentile of Chl-a for at least three consecutive days. A similar method has been used in previous studies to characterize compound MHWs and extreme Chl-a events^51,55^ or standalone extremes^71^. The selection of these thresholds was also based on the rationale described in the previous section of the Methods (“Marine heatwaves/ marine cold-spells”). Thresholds and durations should be adjusted based on the specific scientific question^6,8,50^ and, in our case, the specific variable (Chl-a concentration). Our choice focused primarily on the minimum number of days (3 days) needed to validly represent the average presence of phytoplankton biomass (before it is grazed, sunk, etc.)^133,134^, while aiming to better capture phytoplankton responses to extreme SST events. The daily Chl-a climatology and thresholds were constructed in the same manner as the temperature climatology and thresholds of the MHWs (MCSs).

### Software used for analysis and visualization

All analyses were conducted using the MATLAB software (version R2021b). The final versions of all figures were created using the open-source raster graphics editor GIMP (The GIMP Development Team, 2019).

## Electronic supplementary material

Below is the link to the electronic supplementary material.

Supplementary Material 1

Supplementary Material 2

## Data Availability

The SST data sets derived from the CMEMS from the “Global Ocean OSTIA Sea Surface Temperature and Sea Ice Reprocessed” are available at: 10.48670/moi-00168. The regionally-tuned Chl-a dataset is based on the European Space Agency’s Ocean Colour Climate Change Initiative (ESA OC-CCI), available at: https://www.oceancolour.org/portal/, and further processed using a regional ocean colour algorithm described by Brewin et al.^117^. The *in situ* based Chl-a data were obtained from the NASA SeaBASS website (http://seabass.gsfc.nasa.gov/. The Argo-float data were collected and made freely available by the International Argo Program and the national programs that contribute to it (https://argo.ucsd.edu ,https://www.ocean-ops.org).
