# Supplementary material for: Response of Red Sea phytoplankton biomass to marine heatwaves and cold-spells
Source: Sci Rep. 2025 Feb 11;15:5109. doi: 10.1038/s41598-025-88727-5 (PMC11814131; doi:10.1038/s41598-025-88727-5)
Supplement: Supplementary file 1 — Supplementary Material 1 [file 41598_2025_88727_MOESM1_ESM.docx]

**Table S1**: Key features of MHWs per region (Northern Red Sea – NRS, North Central Red Sea – NCRS, South Central Red Sea – SCRS, Southern Red Sea – SRS) during the respective winter phytoplankton blooming periods (NRS: January to March, NCRS and SCRS: December to February, SRS: October to January), presented in Figs. 2 and S3 (upper panels). Reference period: 1998 – 2018. MHW detection thresholds: climatological 92nd percentile and a minimum duration of ten days. Rows in bold correspond to cases of compounds with the hypothesized phytoplankton response to the respective SST extreme (i.e., MHW – LChl-a). In some cases, the extreme extends beyond the defined blooming period (e.g., NRS, 2^nd^ blooming period).

| Blooming period serial number | Start Year | Start Month | Start Day | End Year | End Month | End Day | Duration (Days) | Max Intensity (^o^C) | Mean Intensity (^o^C) | Intensity Variance (^o^C) | Cumulative Intensity (^o^C) |
| --- | --- | --- | --- | --- | --- | --- | --- | --- | --- | --- | --- |
| NRS |  |  |  |  |  |  |  |  |  |  |  |
| **1** | **2010** | **1** | **4** | **2010** | **3** | **19** | **75** | **2.70** | **1.40** | **0.44** | **104.92** |
| 2 | 2010 | 11 | 5 | 2011 | 1 | 17 | 74 | 2.33 | 1.49 | 0.48 | 110.25 |
| **3** | **2018** | **3** | **18** | **2018** | **3** | **29** | **12** | **1.50** | **1.28** | **0.14** | **15.38** |
| NCRS |  |  |  |  |  |  |  |  |  |  |  |
| 1 | 2006 | 1 | 6 | 2006 | 1 | 15 | 10 | 1.15 | 0.99 | 0.14 | 9.92 |
| 2 | 2010 | 2 | 14 | 2010 | 2 | 26 | 13 | 1.95 | 1.75 | 0.21 | 22.74 |
| 3 | 2010 | 11 | 5 | 2010 | 12 | 12 | 38 | 1.62 | 1.26 | 0.21 | 47.89 |
| 3 | 2010 | 12 | 23 | 2011 | 1 | 8 | 17 | 1.02 | 0.91 | 0.08 | 15.48 |
| 3 | 2011 | 2 | 1 | 2011 | 2 | 11 | 11 | 1.47 | 1.15 | 0.17 | 12.65 |
| SCRS |  |  |  |  |  |  |  |  |  |  |  |
| **1** | **2017** | **1** | **3** | **2017** | **2** | **7** | **36** | **1.28** | **0.93** | **0.20** | **33.54** |
| SRS |  |  |  |  |  |  |  |  |  |  |  |
| **1** | **1998** | **10** | **14** | **1998** | **10** | **27** | **14** | **1.30** | **1.09** | **0.14** | **15.32** |
| **2** | **2015** | **12** | **16** | **2015** | **12** | **25** | **10** | **0.85** | **0.75** | **0.08** | **7.46** |
| **3** | **2017** | **10** | **1** | **2017** | **10** | **28** | **28** | **1.75** | **1.17** | **0.29** | **32.72** |

**Table S2**: Key features of MCSs per region (Northern Red Sea – NRS, North Central Red Sea – NCRS, South Central Red Sea – SCRS, Southern Red Sea – SRS) during the respective winter phytoplankton blooming periods (NRS: January to March, NCRS and SCRS: December to February, SRS: October to January), presented in Figs. 2 and S3 (upper panels). Reference period: 1998 – 2018. MCS detection thresholds: climatological 8^th^ percentile and a minimum duration of ten days. Rows in bold correspond to cases of compounds with the hypothesized phytoplankton response to the respective SST extreme (i.e., MCS – HChl-a). In some cases, the extreme extends beyond the defined blooming period (e.g., NRS, 3^rd^ blooming period).

| Blooming period serial number | Start Year | Start Month | Start Day | End Year | End Month | End Day | Duration (Days) | Max Intensity (^o^C) | Mean Intensity (^o^C) | Intensity Variance (^o^C) | Cumulative Intensity  (^o^C) |
| --- | --- | --- | --- | --- | --- | --- | --- | --- | --- | --- | --- |
| NRS |  |  |  |  |  |  |  |  |  |  |  |
| **1** | **2000** | **2** | **26** | **2000** | **3** | **31** | **35** | **-1.32** | **-1.08** | **0.12** | **-37.77** |
| **2** | **2001** | **2** | **15** | **2001** | **3** | **1** | **15** | **-1.14** | **-0.93** | **0.10** | **-13.99** |
| **3** | **2006** | **12** | **14** | **2007** | **1** | **27** | **45** | **-1.41** | **-1.07** | **0.12** | **-48.34** |
| **4** | **2008** | **2** | **18** | **2008** | **3** | **1** | **13** | **-1.09** | **-0.95** | **0.07** | **-12.37** |
| **5** | **2012** | **1** | **21** | **2012** | **2** | **9** | **20** | **-1.04** | **-0.90** | **0.08** | **-17.91** |
| NCRS |  |  |  |  |  |  |  |  |  |  |  |
| **1** | **2001** | **2** | **7** | **2001** | **2** | **27** | **21** | **-1.31** | **-1.12** | **0.13** | **-23.62** |
| **2** | **2006** | **12** | **29** | **2007** | **1** | **26** | **29** | **-1.25** | **-1.06** | **0.13** | **-30.62** |
| **3** | **2008** | **2** | **20** | **2008** | **2** | **29** | **10** | **-1.52** | **-1.08** | **0.23** | **-10.78** |
| **4** | **2011** | **11** | **19** | **2011** | **12** | **20** | **32** | **-1.48** | **-1.22** | **0.17** | **-39.06** |
| SCRS |  |  |  |  |  |  |  |  |  |  |  |
| 1 | 2001 | 2 | 15 | 2001 | 2 | 26 | 12 | -0.88 | -0.83 | 0.04 | -9.96 |
| **2** | **2005** | **1** | **10** | **2005** | **1** | **26** | **17** | **-0.80** | **-0.71** | **0.04** | **-12.15** |
| **3** | **2006** | **12** | **31** | **2007** | **1** | **19** | **20** | **-1.05** | **-0.81** | **0.13** | **-16.22** |
| **4** | **2008** | **1** | **31** | **2008** | **2** | **9** | **10** | **-1.26** | **-0.91** | **0.23** | **-9.07** |
| **4** | **2008** | **2** | **16** | **2008** | **2** | **29** | **14** | **-0.95** | **-0.82** | **0.08** | **-11.49** |
| 5 | 2011 | 11 | 22 | 2011 | 12 | 13 | 22 | -1.20 | -0.87 | 0.19 | -19.23 |
| SRS |  |  |  |  |  |  |  |  |  |  |  |
| 1 | 2004 | 10 | 31 | 2004 | 11 | 22 | 23 | -1.57 | -1.17 | 0.19 | -26.88 |
| **1** | **2004** | **11** | **26** | **2004** | **12** | **12** | **17** | **-1.37** | **-0.80** | **0.22** | **-13.62** |
| **2** | **2006** | **1** | **3** | **2006** | **1** | **14** | **12** | **-1.10** | **-0.82** | **0.18** | **-9.89** |
| **3** | **2009** | **10** | **25** | **2009** | **11** | **4** | **11** | **-1.44** | **-1.29** | **0.09** | **-14.14** |
| **4** | **2010** | **12** | **10** | **2010** | **12** | **20** | **11** | **-0.84** | **-0.67** | **0.10** | **-7.42** |
| **4** | **2010** | **12** | **30** | **2011** | **1** | **11** | **13** | **-1.28** | **-0.97** | **0.19** | **-12.55** |
| **5** | **2012** | **10** | **9** | **2012** | **10** | **27** | **19** | **-1.90** | **-1.36** | **0.36** | **-25.90** |
