# Supplementary material for: Response of Red Sea phytoplankton biomass to marine heatwaves and cold-spells
Source: Sci Rep. 2025 Feb 11;15:5109. doi: 10.1038/s41598-025-88727-5 (PMC11814131; doi:10.1038/s41598-025-88727-5)
Supplement: Supplementary file 2 — Supplementary Material 2 [file 41598_2025_88727_MOESM2_ESM.docx]

**SUPPLEMENTARY MATERIAL**


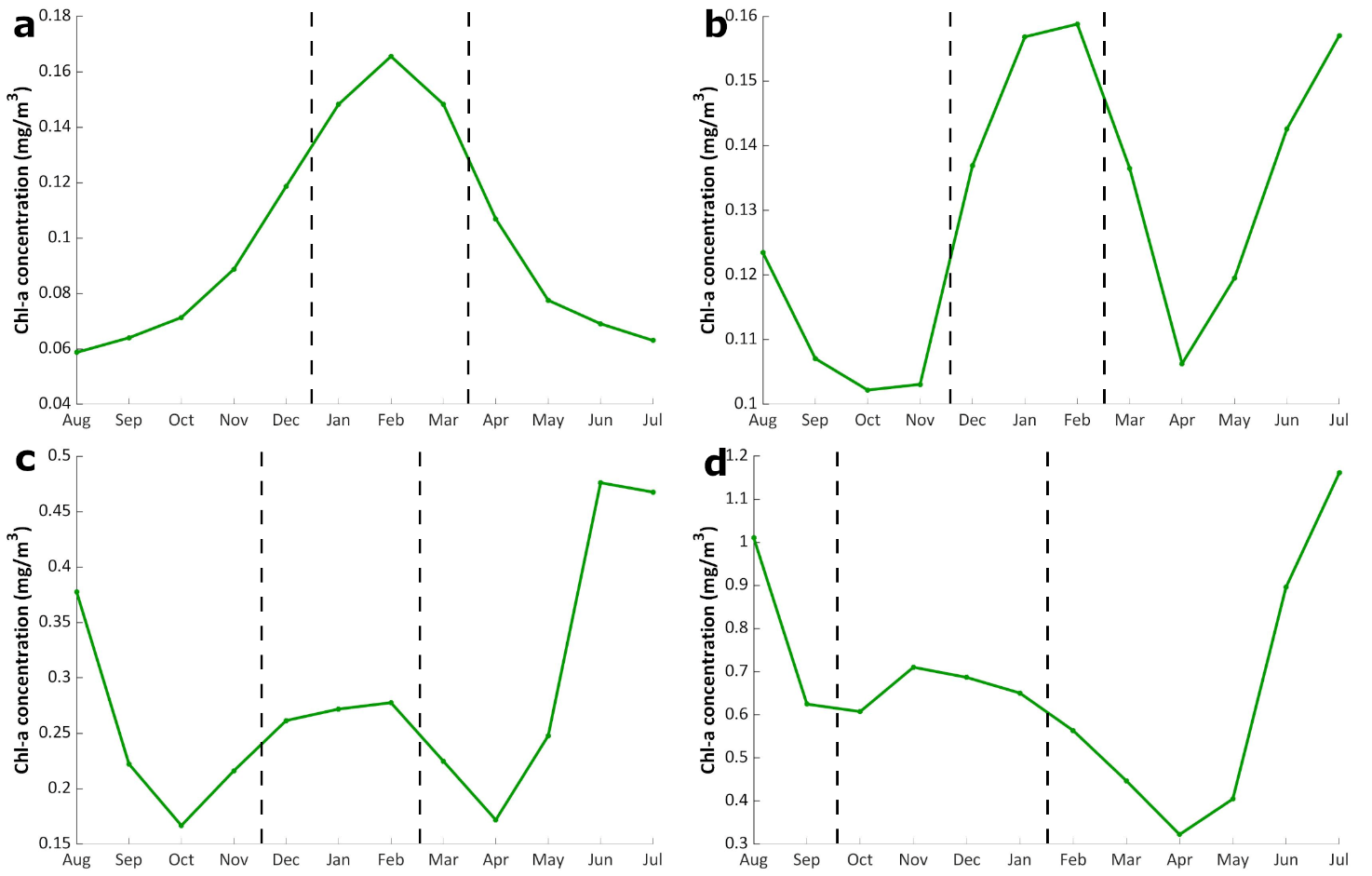


**Figure S1. Monthly climatological averages of Chl-a concentration (mg/m^3^).** **a)** Northern Red Sea, **b)** North Central Red Sea**, c)** South Central Red Sea, **d)** Southern Red Sea, based on the reference period 1998-2018. Vertical dashed lines indicate the winter phytoplankton growth period per region.


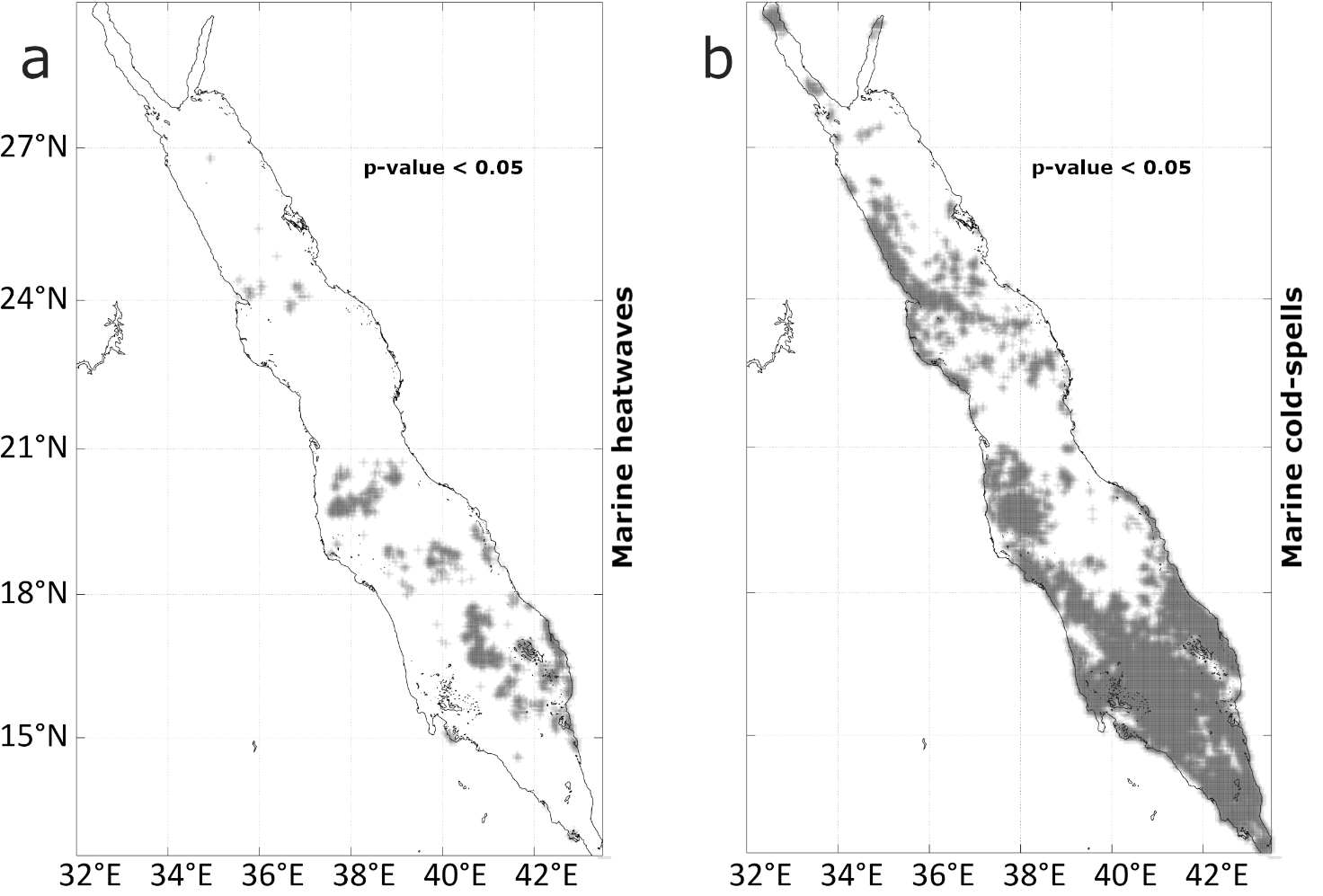


**Figure S2**. **P-values of decadal trends in the total number of days of MHW and MCS recorded during winter Chl-a blooming periods (October-March), in the Red Sea for the years 1982-2018**. The analysis is based on satellite-derived SST data. **(a)** Crosses indicate areas where the p-value of MHW trend is lower than 0.05. **(b)** Crosses indicate areas where the p-value of MCS trend is lower than 0.05.


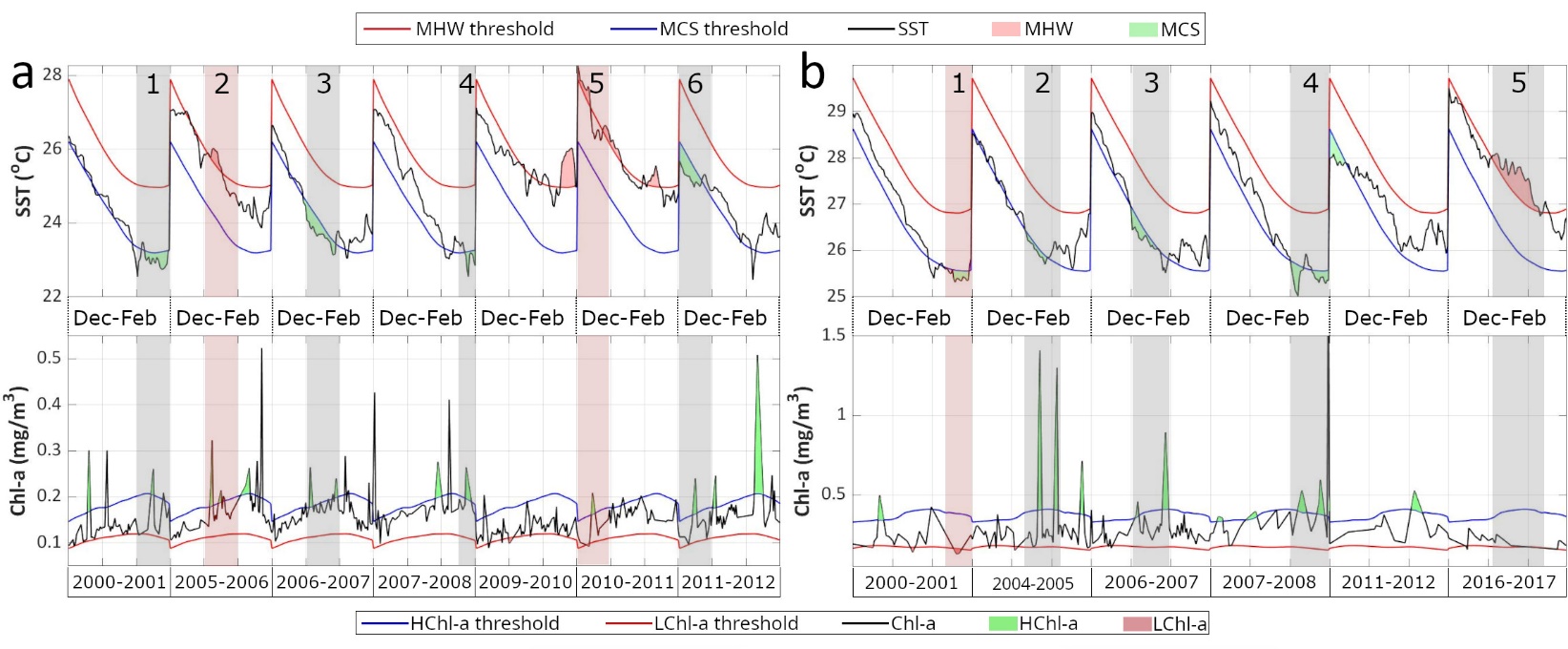


**Figure S3.** **SST and Chl-a timeseries, of annual winter Chl-a blooming periods (December to February) including extreme SST events (MHW/MCS) and coinciding extreme Chl-a events (LChl-a/HChl-a).** **(a)** North Central Red Sea. **(b)** South Central Red Sea. In both panels the upper section concerns SST (C^o^): black line represents daily spatially-averaged satellite-derived SST values, red line denotes the daily 92^nd^ percentile value of SST, blue line depicts the daily 8^th^ percentile value of SST, and red/green shaded areas correspond to MHWs/MCSs. The lower section presents Chl-a concentration (mg/m^3^): black line signifies daily spatially-averaged satellite-derived Chl-a concentration values, blue line indicates the daily 90^th^ percentile value of Chl-a concentration, red line depicts the daily 10^th^ percentile value of Chl-a concentration, and green/red shaded areas denote HChl-a/LChl-a. Shaded grey regions and respective numbering indicate the concurrent MHW and LChl-a, or MCS and HChl-a events . Shaded light-red regions show compound events that fail our hypothesis (e.g. MHW and HChl-a).


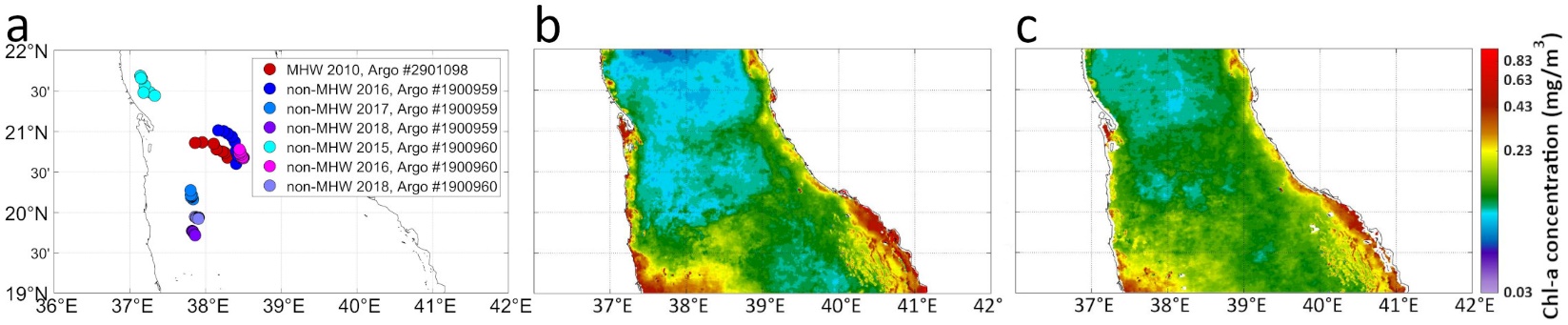


**Figure S4.** **MHW event (7/11 – 3/12/2010), in the South Centrals Red Sea (SCRS); Argo-floats trajectories, and surface Chl-a concentration (mg/m^3^) comparison between the MHW period and non-MHW Years (7/11 – 3/12, Years 2015-2018).** **(a)** Argo-floats trajectories during the MHW (red circles; 7/11 – 3/12/2010) and during same period of non-MHW years (7/11 – 3/12, Years 2015-2018). Satellite-derived data showing surface Chl-a concentration during **(b)** the MHW and **(c)** non-MHW years.


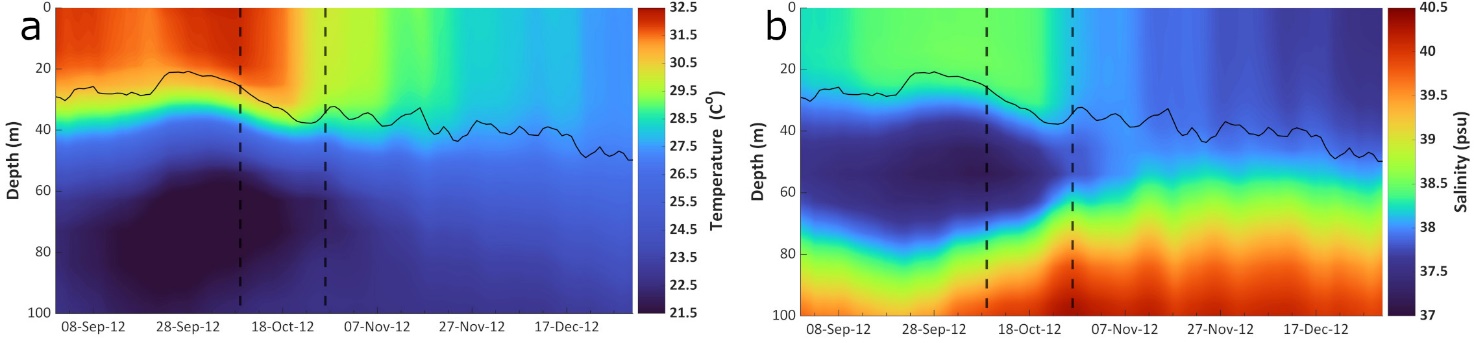


**Figure S5.** **Physical characteristics of the water column of the Southern Red Sea (SRS), before, during and after a MCS event (9/10 – 27/10/2012) during winter Chl-a blooming period (October to January, 2011-2012).** Model outputs of spatially averaged vertical profiles of **a)** temperature and **b)** salinity and MLD; vertical dashed lines indicate the MCS period.
